# Nonthermal Effect of Microwave Processing Enhances Interface Reactivity and Microchannel Integrity: Low-Temperature Rapid Bonding of PMMA Microfluidic Devices

**DOI:** 10.1021/acsomega.4c07013

**Published:** 2024-12-18

**Authors:** Shu-Cheng Li, Jian-Ruei Chen, Chao-Ching Chiang, Yi-Sung Tsai, Benjamin Tien-Hsi Lee

**Affiliations:** † Department of Mechanical Engineering, National Central University, Taoyuan, Taiwan 320317, Republic of China; ‡ Department of Mechanical Engineering, Asia Eastern University of Science and Technology, New Taipei City, Taiwan 220303, Republic of China

## Abstract

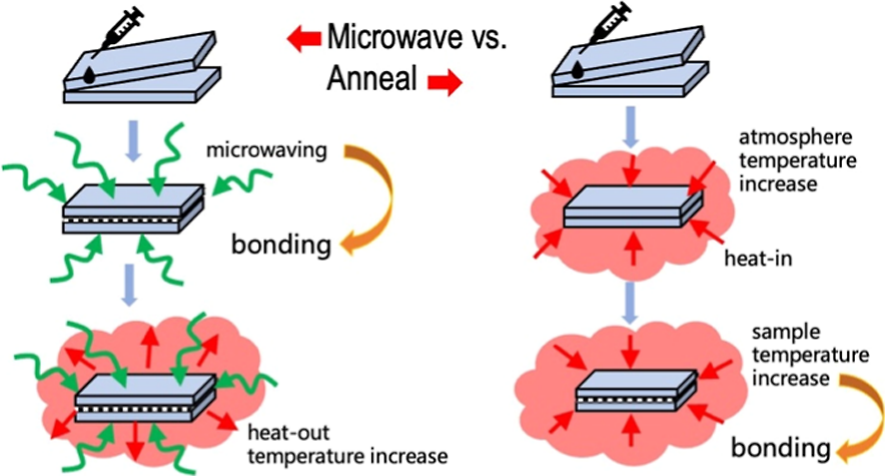

As a nonthermal approach, microwave processing significantly enhances interface reactivity and preserves microchannel integrity during the bonding of poly(methyl methacrylate) (PMMA) microfluidic devices. By activating and aligning polymer chains at lower temperatures, this method promotes rapid bonding and improved interfacial adhesion, maintaining the precision of delicate microstructures essential for device functionality. Unlike thermal wafer bonding, which relies on elevated temperatures that may risk deforming delicate microstructures, the nonthermal effect of microwaves facilitates the activation and alignment of polymer chains at lower temperatures, enhancing interfacial adhesion through improved molecular interactions. Comprehensive experiments employing X-ray photoelectron spectroscopy and atomic force microscopy revealed that microwave treatment significantly improved the surface reactivity of PMMA, resulting in a bond strength that surpassed that of traditional methods without reaching the thermal degradation threshold. The rapid evaporation of isopropanol under microwave exposure minimizes thermal buildup, further demonstrating the contribution of nonthermal microwave effects to the bonding process. This approach represents a breakthrough in microfluidic device fabrication, balancing effective bonding with structural integrity, and holds significant promise for applications in biomedical engineering and MEMS.

## 1 Introduction

In the field of microelectromechanical systems (MEMS), recent technological advancements have significantly enhanced the miniaturization and efficiency of biomedical engineering applications. Innovations in semiconductor technology are particularly crucial for the development of compact wafers, which are essential for realizing MEMS functionality. These advancements are especially important for enhancing biomedical systems by integrating microchannels with state-of-the-art separation and filtration technologies, aiming to overcome the limitations of traditional biomedical testing methods. Conventional methods are often time-consuming, require large sample volumes, and demand substantial financial and human resources. Microfluidics, an interdisciplinary field involving microelectronics, new materials, chemistry, biology, and biomedical engineering,^1^ stands out because of its high precision, low cost, and efficiency. This technology is applicable not only in medicine but also in chemical synthesis,^2^ cell biology,^3^ and environmental monitoring.^4^

Traditionally, microchannel fabrication materials have been primarily inorganic, such as glass and silicon, which are gradually being phased out owing to their high brittleness, processing difficulties, and high costs. In contrast, polymer materials are now widely used because of their low cost, ease of processing, and high chemical stability.^5^ These polymer materials^6^ include polycarbonate (PC), cyclic olefin copolymer (COC), cyclic olefin polymer (COP), polyethylene (PE), polypropylene (PP), polydimethylsiloxane (PDMS), and poly(methyl methacrylate) (PMMA).

Several key application areas for bonded microfluidic devices are identified.

1. Biomedical diagnostics:^7,8^ the bonded PMMA microfluidic devices are ideal for lab-on-a-chip applications in point-of-care diagnostics like blood testing, DNA analysis, and sample preparation, enabling rapid onsite diagnosis without large laboratory equipment. The strong bond integrity ensures reliable handling of biological samples in cell analysis and drug testing, providing sealed, contamination-free microchannels.
2. MEMS and sensor applications:^9,10^ this bonding method is applicable to MEMS fabrication, preserving delicate components while providing durable bonds. It also supports sensor development for detecting chemical or biological analytes in industrial and medical applications, ensuring structural integrity during operation.
3. Drug delivery systems:^11,12^ the bonded microchannels are suitable for controlled drug delivery systems, ensuring precise and consistent medication administration over time, which is critical in medical settings.
4. Research and development platforms: the bonding technique offers a low-cost, reliable, and scalable solution for fabricating microfluidic platforms used in research across fields like chemistry, biotechnology, and beyond.

Bonding methods^10,13−15^ for polymers can be classified into direct bonding methods and indirect bonding methods. Indirect bonding uses an intermediate material^16^ to bond two substrates together. Direct bonding, on the other hand, does not require additional materials at the interface, as the polymer itself can act as an adhesive under certain conditions, forming microchannels with homogeneous sidewalls. Direct bonding involves sealing the substrates via thermal,^17^ microwave,^18,19^ acoustic,^20^ mechanical,^21^ or chemical energy,^15^ or several energies acting together.^22^ The most traditional direct bonding method is thermal compression bonding, which heats the substrates to near or above the glass transition temperature of one or both substrate materials and applies pressure. This causes the polymer chains at the molten surfaces to interdiffuse, followed by annealing to form a strong bond. Although this method has advantages such as simplicity, high bonding strength, and uniformity, issues such as thermal deformation and thermal stress become significant as microchannels become more refined. Additionally, the poor thermal conductivity of polymers results in long processing times.

Currently, several methods have been developed for bonding PMMA substrates, including infrared-assisted bonding,^23^ laser heating,^24^ ultrasonic-assisted bonding,^20,25,26^ solvent bonding,^15,27,28^ and microwave wafer bonding.^18,19,29,30^ These approaches are based on thermal treatment, whether by infrared, laser, annealing, or microwave methods, to fuse the bonding interface via a typical solvent-based bonding approach. Importantly, controlling the solvent dosage in solvent bonding is challenging; excess solvent can erode microchannels, whereas insufficient solvent reduces the bonding area. Consequently, among these methods, microwave-assisted solvent bonding^19,29,30^ has emerged, even when a simple household microwave oven is used.

However, the nonthermal effect of microwaves also plays a critical role, alongside the thermal effect, in inducing chemical reactions at the PMMA bonding interface and the solvent. The nonthermal effect arises from the direct interactions of electromagnetic field energy with specific molecules, ions, or species, particularly those with polarity, within the reaction system, independent of the macroscopic temperature effect.^31,32^ Even nonthermal effects can accelerate organic chemical reactions in a sufficient low-temperature environment.^33,34^ We believe that this dual action of nonthermal and thermal effects modifies the surface and allows the addition of a small amount of solvent to achieve high-strength bonds without damaging the microchannels.

In this study, isopropanol (IPA) was used as the solvent, and microwave assistance was used to bond the substrates. The aim was to investigate the mechanism of microwave-assisted solvent bonding and to explore the optimal microwave time for this bonding process. We demonstrated the effectiveness of this approach for bonding PMMA microfluidic devices, particularly for microchannels with widths and depths of 500 μm. Recent advancements suggest that with precise control of solvent concentration, microwave exposure time, and microchannel design, this technique could potentially be adapted to microchannels as small as 100 μm.^35^ For instance, Zhang et al. achieved minimal distortion in PMMA channels as small as 80 μm × 80 μm by optimizing solvent bonding parameters,^20^ while Ng et al. demonstrated that vacuum-assisted bonding effectively reduced distortion in channels as small as 200 μm.^36^ This method emphasizes the seamless integration of theoretical microwave principles with practical design strategies, heralding a new era of efficient microfluidic device fabrication through microwave-assisted solvent bonding.

The microwave-assisted solvent bonding method offers significant advantages in scalability, cost-effectiveness, and versatility, making it ideal for industrial-scale production. This technique, using common microwave ovens, enables high-throughput manufacturing by processing multiple devices simultaneously, increasing production efficiency. With low energy consumption and minimal solvent use (e.g., IPA), this method reduces operational costs and aligns with sustainable practices. Its ability to bond various thermoplastics, such as PMMA, PC, and COC, broadens its application across industries such as microfluidics, biomedical diagnostics, and chemical processing. Rapid bonding times (100–140 s) and strong adhesion ensure device reliability, making this method a practical and eco-friendly solution for large-scale production. The key industrial advantages include the following:

(1) Scalability: microwave methods allow easy adaptation to large-scale, high-throughput production; (2) low cost: reduced energy use, minimal material degradation, and no need for expensive tools; (3) faster production: shorter bonding times than traditional methods; (4) versatility: applicability to various thermoplastics, meeting diverse industrial needs; (5) reliability: durable bonds suitable for intricate microchannel designs; and (6) eco-friendly: minimal solvent use reduces chemical waste and aligns with sustainable practices.

## 2 Experimental Methods

### 2.1 Materials and Reagents

The test samples were purchased from Tsuyang Company, Taiwan, and consisted of 100% methyl methacrylate monomer (CM-250X) as the base material. The test samples were protected by a low-viscosity protective film on the surface to prevent surface scratches during transportation. Therefore, before the experiment begins, the test samples were cleaned to remove any residual adhesive or contaminants between the samples and the protective film. The primary solvent used in the experiment was industrial-grade IPA (purity: 95%). IPA was chosen because its Hildebrand solubility parameter (δ: 23.8) was highly similar to that of PMMA (δ: 19).

### 2.2 Microchannel Fabrication

**Figure 1:**
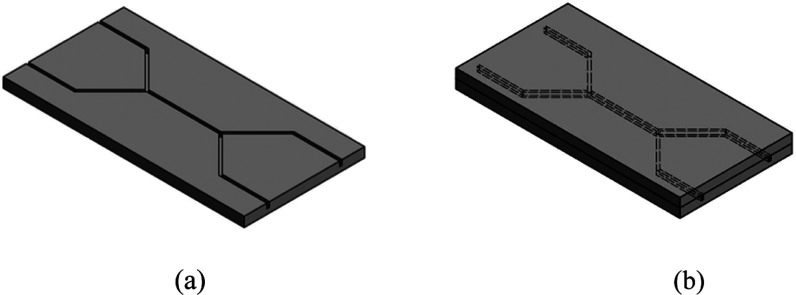
Design of the microfluidic chip: (a) schematic of the microfluidic design and (b) bonded model of the microfluidic test pieces.

PMMA materials exhibit excellent micromilling performance, allowing for the fabrication of microchannels through laser cutting. First, PMMA test samples with dimensions of 50 mm × 25 mm × 2 mm were fixed on the platform of the laser cutting machine. The microchannels, with a width of 0.5 mm and a depth of 0.5 mm, as shown in Figure 1a, were then cut. Postprocessing was required to address the slight deformation caused by the laser on the microchannel surfaces. The surface was polished via a regular polishing machine with a polishing slurry composed of 1 μm aluminum oxide powder and water (weight ratio: 1:10). A smooth, mirror-like surface facilitates the subsequent bonding process.

### 2.3 Adhesive Strength Test

**Figure 2:**
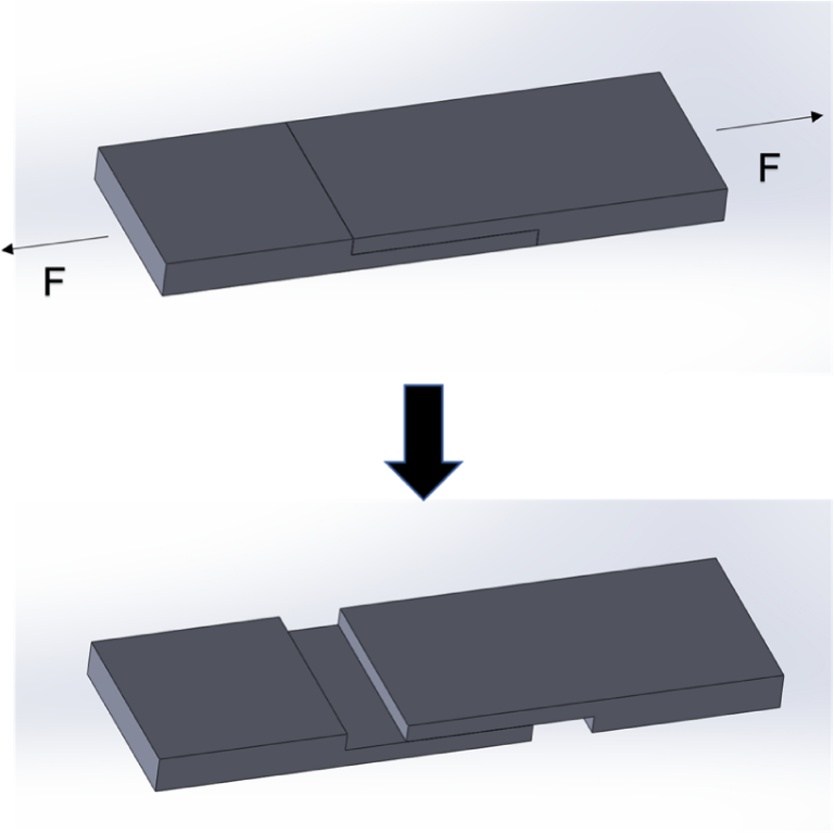
Tensile test program model.

The adhesive strength was characterized via a tensile test. The tensile testing equipment used was a floor-standing computer-controlled universal testing machine (HT-2402) manufactured by Hung Ta Instrument Co., Ltd., Taiwan. The PMMA test samples were stretched in the manner shown in Figure 2, from which the tensile strength was obtained.

### 2.4 Experimental Procedure

This experiment utilizes microwave solvent bonding, where the nonthermal reactions of polar molecules with the PMMA surface under microwave exposure promote the bonding of the organic solvent at the interface. Before the experiment, the PMMA samples were thoroughly cleaned. The cleaning process involved treating the samples with ethanol, followed by extensive rinsing and ultrasonic cleaning in deionized (DI) water to ensure pristine surface conditions conducive to bonding. The experiment was divided into three main parts. After the experiments were completed, the surface morphology and chemical composition were characterized via AFM and XPS, and the surface morphology of the samples was characterized via optical microscopy. The adhesive strength was evaluated via a tensile testing machine.

### 2.5 Surface Sample Preparation for Detection

At room temperature, a syringe was used to extract the organic solvent from the beaker. Depending on the experimental requirements, the organic solvent was either directly applied to the surface or placed in a microwave oven. After the specified time, the surface of the sample was dried. The sample was then fixed onto a dedicated metal plate for surface detection via the appropriate instruments.

### 2.6 Preparation of Tensile Test Samples and Microchannel Bonding

**Figure 3:**
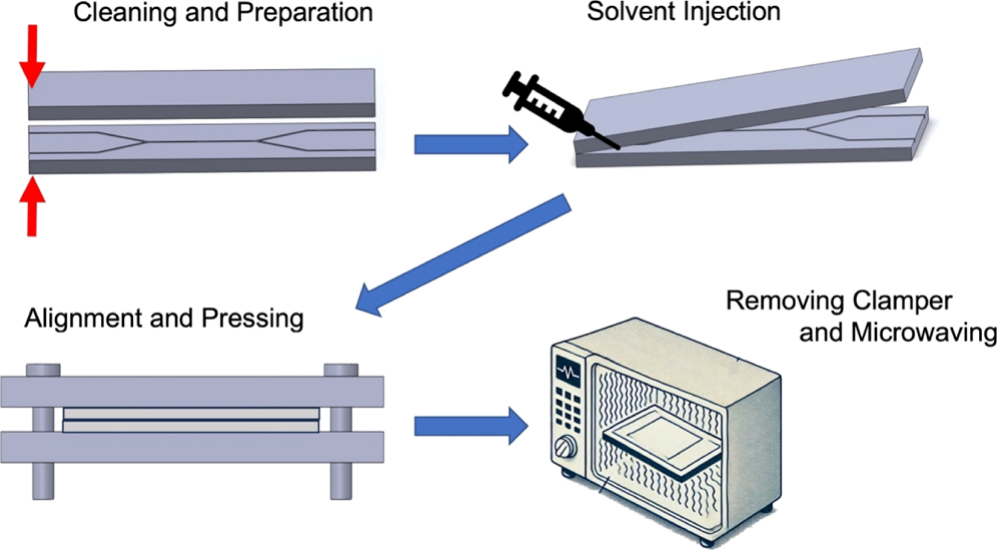
Steps of microwave-assisted solvent bonding of PMMA: (1) cleaning and preparation: PMMA chips are thoroughly cleaned with deionized water to remove contaminants. (2) Solvent application: isopropanol (IPA) is injected at the bonding interface, with the substrates held at a 10° angle for even solvent distribution. (3) Alignment and pressure application: substrates are carefully aligned and pressed together to remove excess solvent and air bubbles. (4) Microwave treatment: bonded substrates are exposed to microwave radiation, triggering a bonding reaction through the nonthermal effects of microwave energy.

As shown in Figure 3, at room temperature, a syringe was used to extract IPA. The two samples to be bonded were positioned with an opening angle of approximately 10°, and a small amount of IPA was carefully injected near the corner via the syringe. The upper plate of the microchannel was then slowly placed over it. During the bonding process of the microchannel samples, care was taken to ensure that no solvent residue remained in the microchannel. We used a syringe to remove any remaining solvent in the microchannel or applied slight pressure to the samples to eliminate any air bubbles at the bonding interface. The samples were clamped before microwaving to ensure proper alignment and initial adhesion during preparation. Clamping helps maintain uniform pressure on the bonding surfaces. However, removing clamps before microwave processing is critical to avoid the risk of fire or equipment damage during the heating process. An infrared temperature sensor is used to monitor the chamber temperature, ensuring that it does not significantly exceed room temperature, thereby preventing experimental errors. After checking, microwaving commenced. Upon completing the bonding process, the samples were dried and then subjected to instrument detection.

## 3 Results and Discussion

### 3.1 AFM Surface Examination of the Materials

This experiment restored the three different stages of the PMMA interface during the experiment, namely, the PMMA was cleaned with water only, treated with isopropyl alcohol (IPA) for 120 s, and microwaved for 120 s after IPA treatment. For surface microscopic morphology, each sample was tested more than five times. Tests were also conducted at different locations on the samples for comparison.

**Figure 4:**
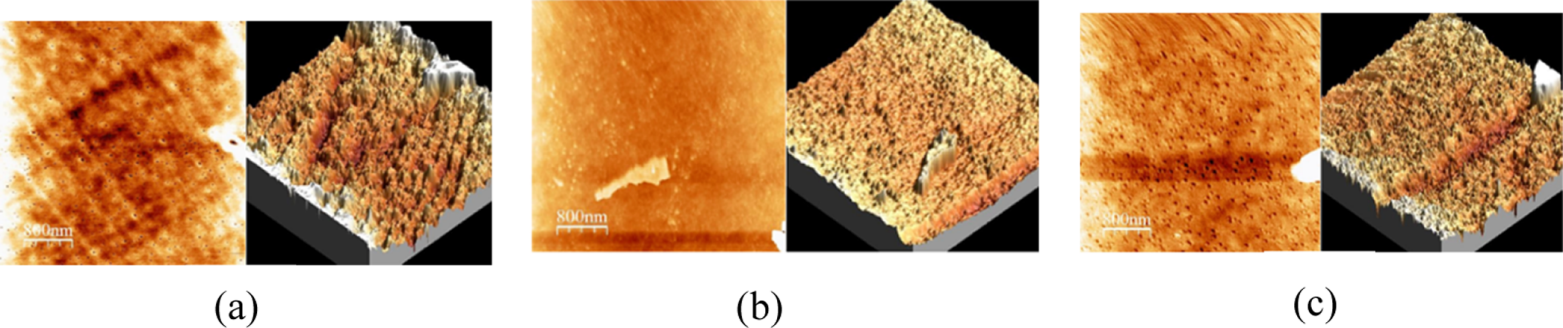
Surface status of the PMMA samples (scale bar = 800 nm) after (a) only water cleaning, (b) only IPA treatment for 120 s, and (c) IPA + microwave treatment for 120 s. AFM 2D and 3D images. These images highlight that the PMMA surface becomes rough after solvent treatment. Notably, the incorporation of microwave assistance resulted in more holes than did the solvent treatment alone.

Figure 4 presents the results characterized by AFM for the three different stages. The three-dimensional image of the sample after cleaning only, as shown in Figure 4a, reveals that the surface of the sample is almost flat. Figure 4b shows that the surface of the sample treated with IPA has many pores, and Figure 4c shows that the sample treated with IPA and microwaved for 120 s has the most pores. This may be due to the contact of the material surface with IPA, which generates tiny pores, making it easier for the IPA to penetrate the undissolved parts of the material. The raised structures on the treated sample may be gel layers or solidified swollen layers formed during the dissolution process of the polymer material. The formation of an appropriate amount of gel and swollen layers is beneficial for adhesion, and the molecules of the gel layer infiltrate the micropores on the surface of the adherent, forming strong mechanical interlocking forces.

**Figure 5:**
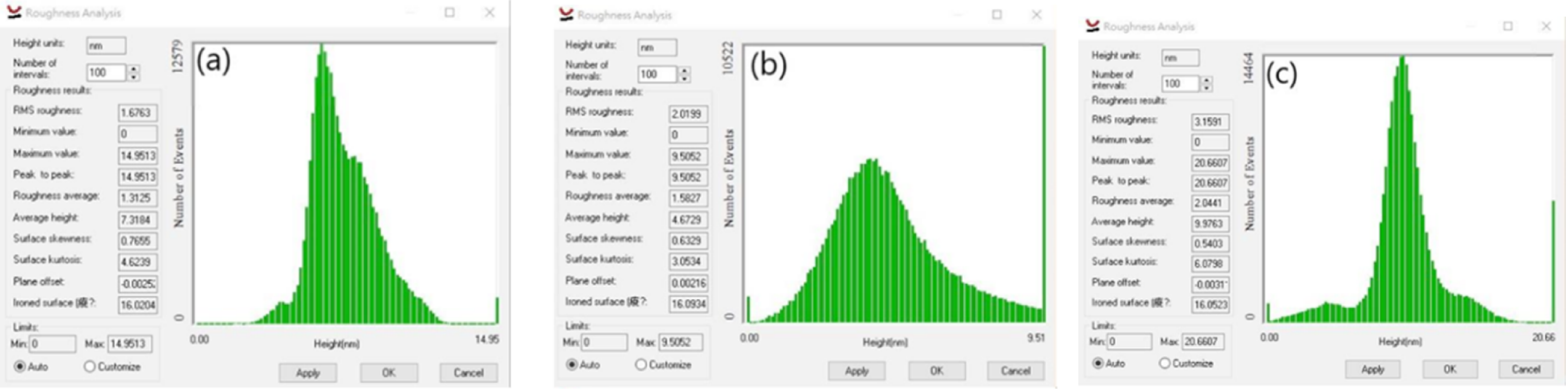
AFM inspection of the PMMA surface status after (a) water cleaning only, (b) IPA treatment for only 120 s, and (c) IPA + microwave treatment for 120 s. These images highlight that the surface roughness and RMS of the PMMA surface increase after solvent treatment. Notably, the surface treated with microwaves exhibited greater roughness and RMS than did the surface treated with solvent alone.

Figure 5 shows the surface state measurements of the three treatment methods mentioned above via AFM. As shown in Figure 5a, the surface roughness and RMS are the lowest after only cleaning, at 1.3 and 1.6 nm, respectively. Figure 5b,c clearly shows that the surface roughness and RMS increase after the application of IPA and further increase after microwaving, with the highest surface roughness and RMS values of 2 and 3.2 nm, respectively, after microwaving. Changes in surface roughness affect surface tension; for solvent-based polymers, surface tension influences the extent to which the solvent wets the sample. The relationship between the adhesion work of the solid or liquid and surface tension is derived from the following equation
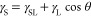
1

The work of adhesion *W*_A_ between the solid and liquid is
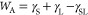
2

Thus, we can derive
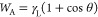
3

According to eq 2, the condition for a liquid to wet the surface of a material is γ_S_γ_L_ > γ_SL_, which is derived from eq 3. Equation 3 shows that the smaller the contact angle is, the greater the adhesion work. The highest surface roughness after the application of IPA, followed by microwaving, implies good surface tension and therefore greater adhesion, allowing IPA to be more evenly distributed on the adhesive surface.

### 3.2 XPS Detection and Analysis

**Figure 6:**
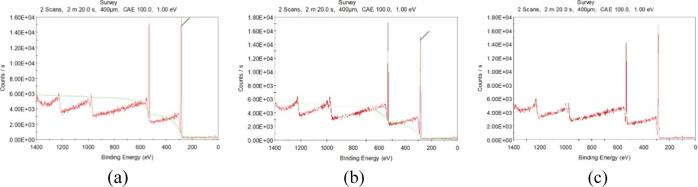
Original XPS spectra of the surface (a) cleaned with water only, (b) treated with IPA only for 120 s, and (c) treated with IPA + microwave for 120 s. Notably, the peak intensity changes after the use of IPA followed by microwave treatment compared with that after the use of IPA alone.

Figure 6 presents the original XPS spectra of the PMMA surface after three different treatments: water cleaning only, IPA treatment, and IPA followed by microwaving. Figure 6a shows the surface state of PMMA after water cleaning. The main peaks correspond to the core levels of the elements present in PMMA, with the highest peaks typically being carbon (C 1s) and oxygen (O 1s). The cleanliness of the surface can be judged by the absence of contaminants or unexpected peaks. Figure 6b shows the spectrum of PMMA after treatment with IPA. The changes in peak intensity indicate the surface chemistry after the solvent interaction. The interaction between IPA and the PMMA surface, as evidenced by changes in peak intensities, suggests that IPA may introduce polar groups that enhance adhesion, thereby increasing surface activation. Figure 6c shows the spectrum after the PMMA surface was treated with IPA followed by microwaving. Compared with the spectrum with IPA treatment only, there are changes in peak intensity, indicating that microwaving induces nonthermal effects on the PMMA surface, intensifying the reaction with IPA. Microwave radiation can enhance the effects of IPA, further activating the surface, which is ideal for wafer bonding processes, as it increases the surface energy and creates more reactive sites.

**Figure 7:**
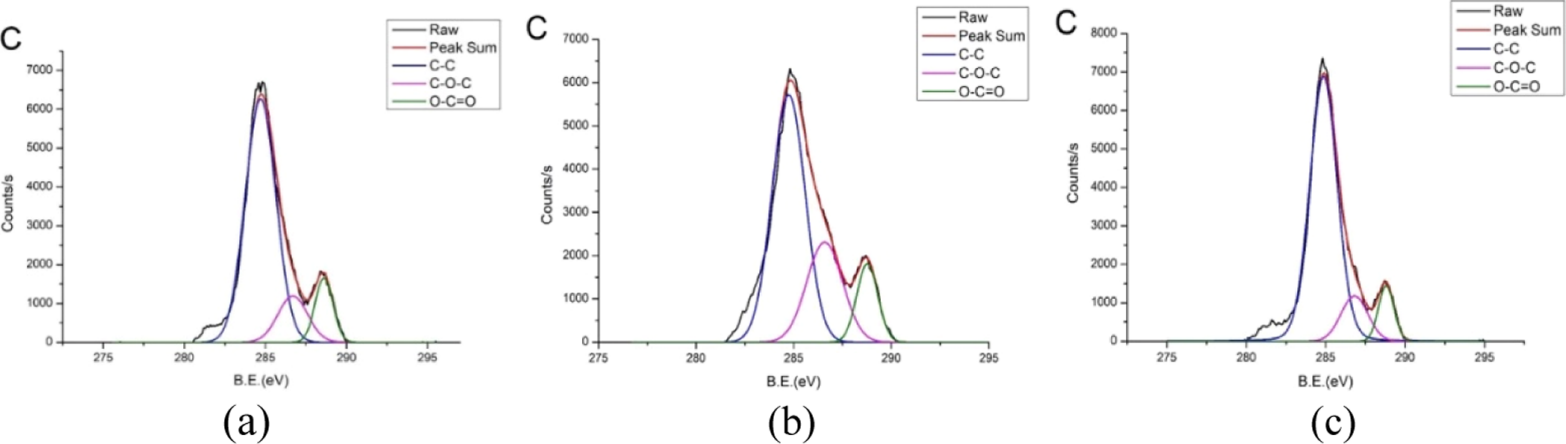
XPS analysis of the narrow carbon discrimination peak results of the surface after (a) water cleaning only, (b) IPA only, and (c) IPA + microwave. Notably, the C–C peak increases after IPA and microwave treatment, whereas the C–O–C and O–C=O peaks both decrease significantly.

Figure 7 presents the XPS analysis of the carbon narrow-scan peaks of the PMMA surface after three treatments: water cleaning only, IPA treatment, and IPA followed by microwaving. The close match between the raw data and the sum of the peak fits indicates that the fitting was accurately performed. Figure 7b shows that the C–O–C peak increases sharply after IPA treatment compared with that in Figure 7a, which reflects only water cleaning. There is a slight decrease in the C–C peak. This change may be due to the contact of IPA with the PMMA material, altering the surface chemical state of the sample. The reaction forms a minor permeation layer, but owing to the adverse effects of the external environmental temperature on continuous solvent permeation, the material generates a solid swollen layer and a gel layer. The solvent remaining in the permeation layer is subsequently consumed and volatilized, causing the disentangled polymer chains to re-entangle and revert to a stable solidified state, leading to changes in the chemical state of the material. A comparison of the XPS results of IPA treatment followed by microwaving with IPA treatment alone revealed that the C–C peak increased by approximately 5%, whereas the C–O–C and O–C=O peaks decreased significantly. This is because microwave irradiation enhances the reaction between IPA and PMMA due to nonthermal effects, causing substantial cleavage of C–O–C and O–C=O bonds and the formation of new bonds.

**Figure 8:**
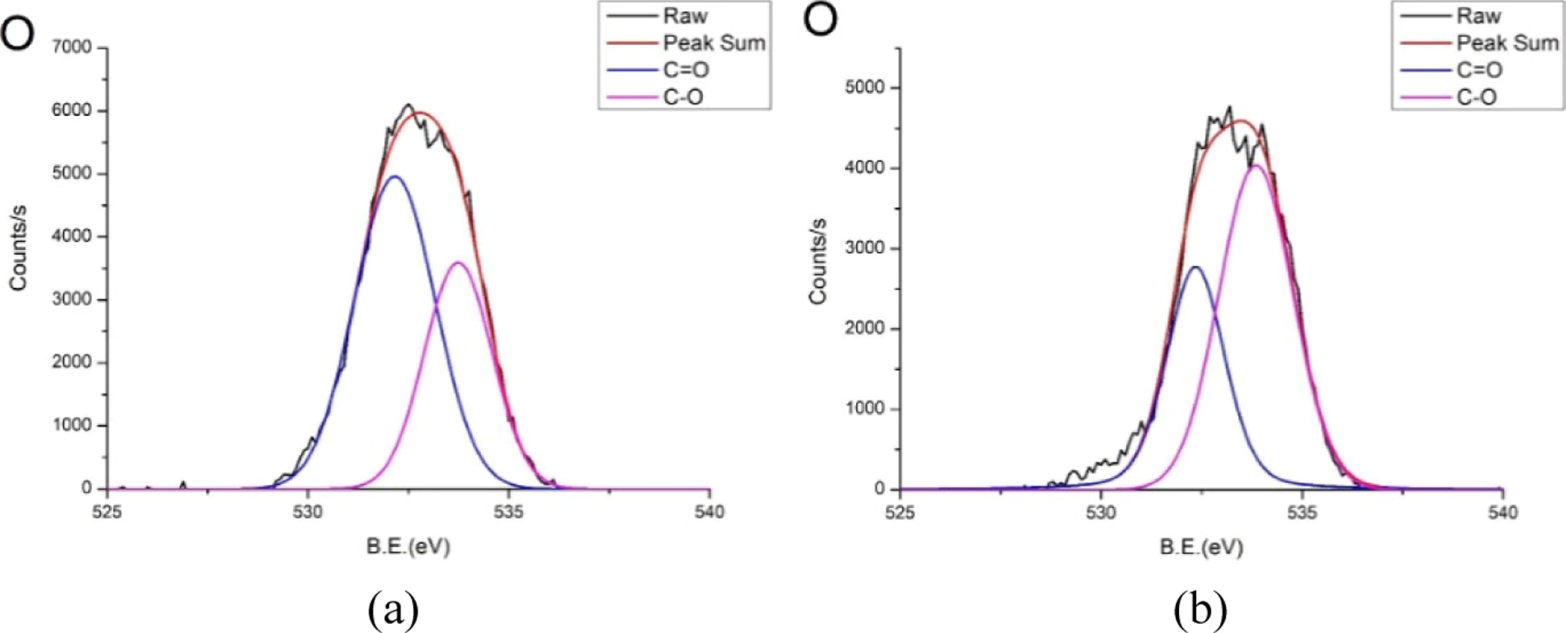
XPS analysis of the narrow oxygen differentiation peak results of surfaces treated with (a) IPA only or (b) IPA + microwave. Notably, the overall oxygen peak decreases after microwave assistance.

Figure 8 shows the XPS analysis results of the oxygen narrow-scan peaks for the PMMA surface treated with IPA only and IPA followed by microwaving. The figure shows that the overall oxygen peak decreases after microwaving. This finding indicates that as microwaving proceeds, nonthermal reactions occur between the sample and IPA, altering the chemical structure of the sample surface. The most significant change is the decrease in the C=O peak and the increase in the C–O peak. This suggests that the surface chemical structure of the PMMA sample forms bonds with IPA. Owing to their varying electronegativity, the carbonyl groups in PMMA undergo bond polarization in a microwave environment, making them susceptible to nucleophilic attack by IPA. During the microwaving process, the carbonyl groups are converted to oxygen bonds with IPA. The chemical structure of IPA includes two single bonds between carbon atoms, which is a key reason for the observed increase in the C–C peak after microwaving.

### 3.3 Tensile Examination of Bonded Specimens

**Figure 9:**
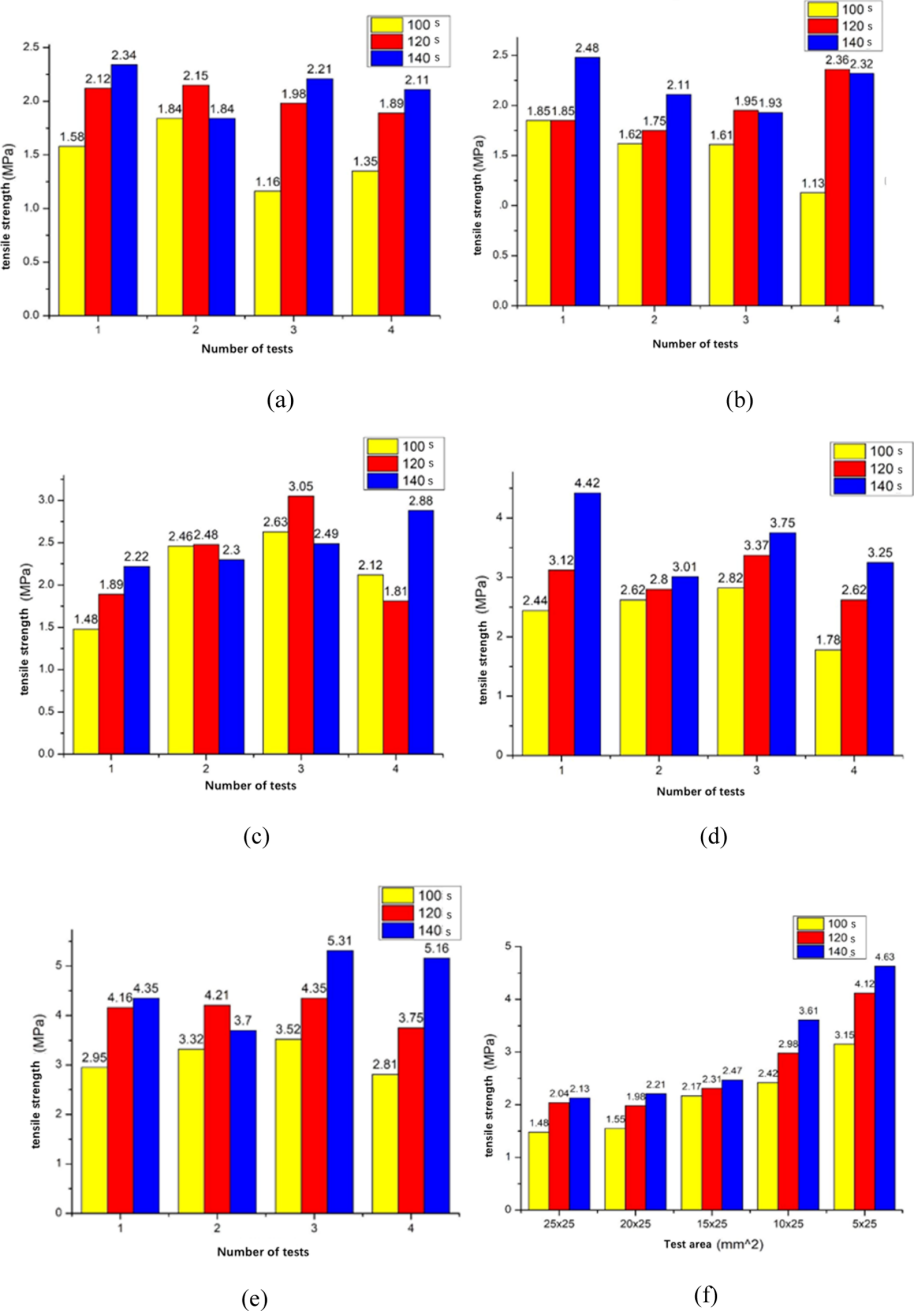
Bonding areas are (a) 25 × 25, (b) 20 × 25, (c) 15 × 25, (d) 10 × 25, (e) 5 × 25 mm^2^, and (f) different area average stretching results. These images highlight the varying tensile strengths of the samples with different bonding areas. Notably, as the bonding area decreases, the bonding strength gradually increases. This suggests that the area of the samples significantly influences the absorption of microwaves during microwave bonding.

**Figure 10:**
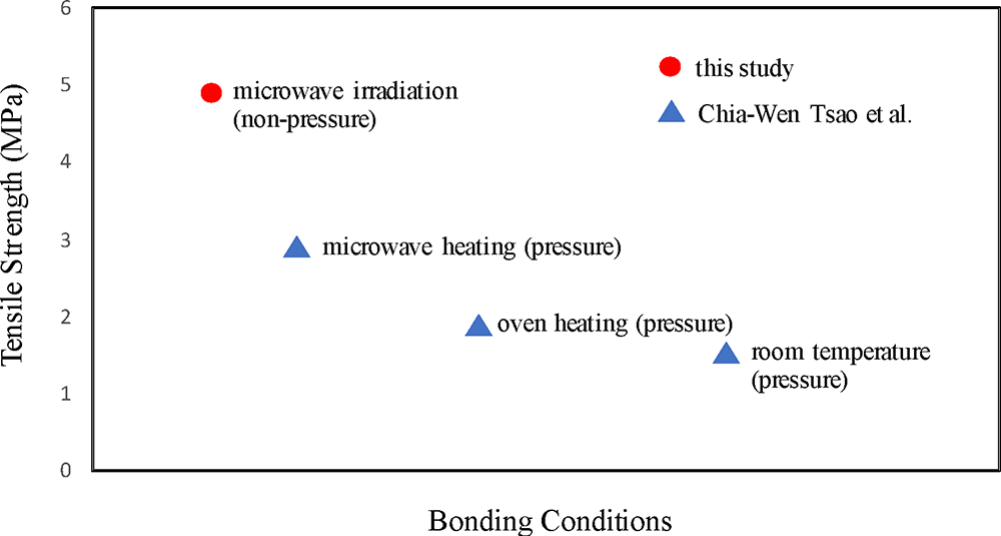
Comparison of bond strength between this study and the study conducted by Chia-Wen Tsao et al.^18^

Figure 9a–e depicts the tensile tests conducted on samples with different bonding areas via a tensile testing machine, which were used to calculate the tensile strength that the bonded areas can withstand. Figure 9f shows a comparison chart of the average tensile data for different areas. Each parameter was tested five times, with the exclusion of extreme values, resulting in four sets of experimental data to avoid errors. The test results indicate that the tensile strength is directly proportional to the microwaving time. For the 25 mm × 25 mm samples, owing to the larger bonding area, the bonding strength is greater, and fractures often occur at parts of the bonding area or the clamped position during tensile testing. Therefore, microwave bonding with a smaller area was performed for comparison. The tensile results can separate the bonding surface as the area shrinks rather than breaking at both ends of the tensile test piece. As shown in Figure 9f, the bonding strength increases gradually with decreasing bonding area. This indicates that the area has a significant effect on the absorption of microwaves during the bonding process. However, smaller areas also increase the likelihood of bonding failure. For small-area bonding, the amount of IPA must be precisely controlled. Excessive IPA can prevent complete volatilization during bonding, resulting in visually observable unbonded areas or solvent residues causing whitening after bonding. When the samples from small areas are removed under low-microwave conditions, unbonded samples can be easily encountered. This is likely due to insufficient time for IPA to interact with the material and form a gel layer, leading to detachment. Therefore, the contact area between IPA and the material surface, as well as microwave absorption, is crucial during the bonding process. Compared with the research conducted by Tsao et al. in 2022,^18^ as shown in Figure 10, under the same conditions, with acetone used as the solvent and without any issues such as microchannel blockage or cracking, this study achieved a superior maximum adhesion strength of 4.83 MPa.

### 3.4 Microchannel Samples under a Microscope

**Figure 11:**
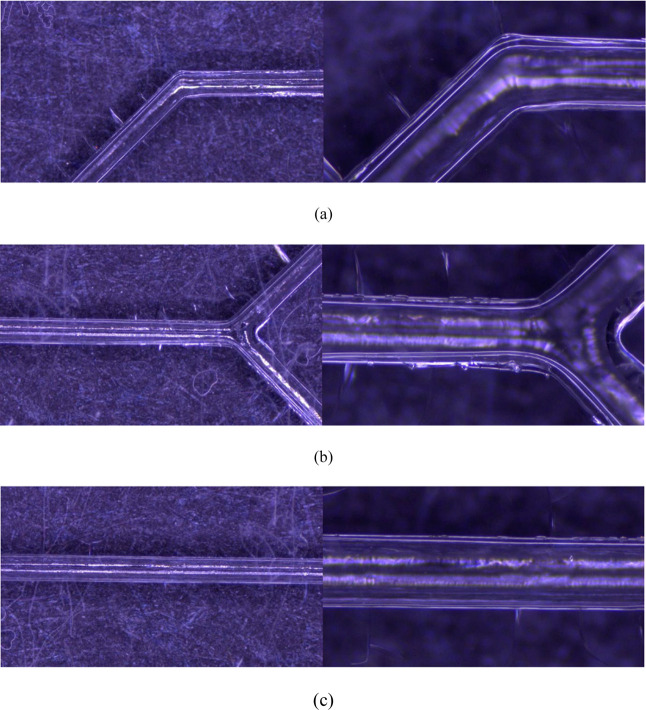
Microwave bonding for a 100 s test piece: (a) bend, (b) liquid intersection, and (c) liquid laminar flow at 50× and 160×. Notably, there are some fine lines perpendicular to the microchannels, and a few air bubbles remain.

**Figure 12:**
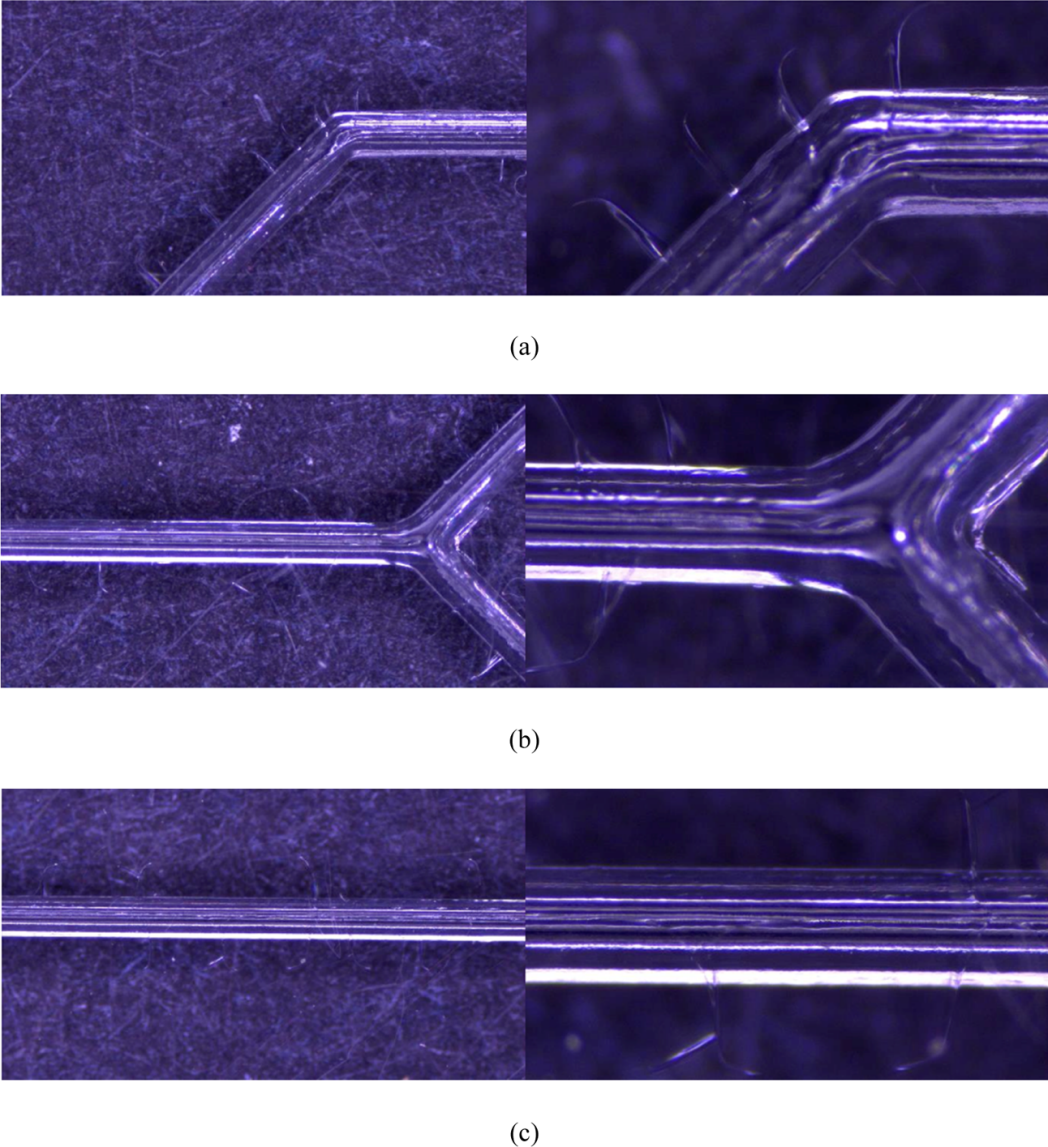
Microwave bonding for a 140 s test piece: (a) bend, (b) liquid intersection, and (c) liquid laminar flow at 50× and 160×. Compared with those at 100 s, the fine lines at the bends are longer and deeper after 140 s of microwave treatment. Additionally, fewer air bubbles remain.

Figures 11 and 12 show the microchannel samples treated with IPA and then microwaved for 100 and 140 s, respectively, and observed under a microscope at magnifications of 50× and 160×. The observations focus on the curved areas, liquid junctions, and laminar flow regions of the microchannels. Vertical fine lines can be observed on the microchannel walls, which are likely due to laser ablation during the laser engraving process. These fine lines are further pronounced when IPA penetrates them during microwave bonding, and temperature changes in the microwave environment deepen these lines. A comparison of the curved areas clearly reveals that the fine lines are longer and deeper at 140 s. Additionally, some residual bubbles are noticeable in the 100 s sample compared with the 140 s sample. This is likely because the gas within the microchannels was not completely expelled when IPA was added to the bonding surface. During the microwaving process, the gas within the microchannels moves to the periphery, affecting the bonding quality. Repeated tests indicate that increasing the microwaving time reduces the residual bubbles in the samples. This could be related to the increase in temperature, which caused the bubbles to diffuse out of the sample through the microchannels as the microwaving time increased.

### 3.5 Inspection of Liquid Injection into Microchannels

**Figure 13:**
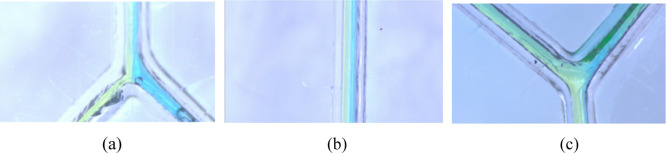
One hundred second test piece liquid injection: (a) liquid intersection, (b) liquid laminar flow, and (c) liquid–end intersection. These images highlight that at both the liquid junction and the laminar flow regions, the two different colored liquids can form laminar flows without mixing with each other. Notably, one side of the liquid mixes at the junction of the liquid ends.

**Figure 14:**
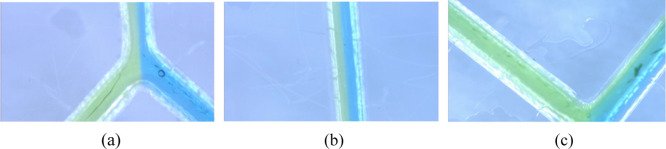
Liquid injection (a) liquid intersection, (b) liquid laminar flow, and (c) liquid–end intersection during 140 s immersion. Compared with 100 s of microwave treatment, the amount of liquid mixing was lower.

Figures 13 and 14 show the results of injecting colored ink into two injection ports after IPA treatment and microwaving for 100 and 140 s, respectively. These observations were made under a microscope at a magnification of 160×, with a focus on the liquid junctions, laminar flow regions, and liquid end junctions. By injecting colored liquids, it is easier to visualize the behavior of the liquid within the microchannels. Under both microwave time parameters, the liquid junctions and laminar flow regions maintain a low Reynolds number, allowing the two colored liquids to form laminar flows without mixing. This demonstrates that the bonding strength of the samples is sufficient to ensure that the microchannels and cover plates are tightly bonded, forming sufficiently small microchannels to achieve a low Reynolds number state. During the experiment, it was also possible to observe the displacement of the laminar flow position under the microscope to determine the increase in the injection volume of a specific solution. At the liquid end junction, however, maintaining the liquid in a laminar flow state was more difficult. Upon passing through the end junction, mixing of one side of the liquid was observed. This mixing could be due to friction between the liquid and the channel walls during flow, residual bubbles in the channels affecting liquid flow, or variations in the channel diameter precision during laser engraving, all of which could cause turbulence at the end junction. The microscopic cracks left in the samples during bonding did not show any liquid flowing into them during the injection process, suggesting that the cracks are located in the cover plate of the sample, thus having minimal impact on the microchannels. Additionally, there was no liquid leakage.

Our study highlights that IPA, as a strongly polar nucleophilic reagent, can facilitate a hydrophilic addition reaction with the carbonyl groups in PMMA, even in the absence of heat generated from water in the solvent. This reaction is driven by the nonthermal effects of microwave radiation, which enhances molecular interactions to form strong bonds without the need for significant thermal energy input. While a moderate temperature increase was observed during the bonding process, this increase is considered a “side effect” resulting from the excitation at the interface between the solvent and the surface. It plays a supplementary role by enhancing solvent evaporation and promoting surface activation but is not the primary driving force behind the bonding process. Specifically, the PMMA surface treated with IPA contains micropores and a gel layer, which are further reinforced by microwave treatment, leading to increased surface roughness and activation. This insight underscores the ability of microwave radiation to induce specific chemical transformations while lowering the activation energy favorable for bonding. Since this study evaluated the effects of microwave treatment on the bonding performance and structural integrity of microfluidic devices under standard operating conditions, aspects such as thermal stability and maximum pressure tolerance, which are crucial for microchannel applications, were not addressed. These issues could be explored in future research.

## 4 Conclusions

In summary, we successfully demonstrated the nonthermal effects of microwave treatment in wafer bonding for PMMA-based microfluidic device fabrication, achieving strong adhesion and structural integrity at low temperatures. The use of 95% IPA is critical for enhancing surface interactions and promoting micropore formation, thereby improving bonding performance and scalability in microfluidic device manufacturing.
